# Mixed epithelial and stromal tumor (MEST) in a young adult male: A case report and literature review

**DOI:** 10.1016/j.amsu.2021.102888

**Published:** 2021-09-22

**Authors:** Muhammad Taha Tariq, Shuah Ullah, Kiran Shafiq Khan, Aneeqa Khan, Irfan Ullah

**Affiliations:** a Dow Medical College, Karachi, Pakistan; b Sindh Institute of Urology and Transplantation SIUT, Karachi, Pakistan; c Kabir Medical College, Gandhara University, Peshawar, 25000, Pakistan

**Keywords:** Tumor, Renal pelvis, Hob nailing, Epithelial, Stromal, MEST, mixed epithelial and stromal tumor, INR, international normalized ratio, TLC, Total leucocyte Count, REST, renal epithelial and stromal tumor, HIV, human immunodeficiency virus

## Abstract

**Introduction and importance:**

A MEST is a rare renal tumor, with stromal as well as epithelial components. It is predominantly benign and local recurrence is not very common. In the majority of the cases, it occurs in females. Its occurrence in a young male makes it a rarity.

**Case presentation:**

A 24 years old male presented at SIUT with the complaint of left flank pain on and off for one month. CT scan showed soft tissue density mass in left renal pelvis extending from mid-pole calyces to pelviuretric junction, leading to obstruction and ultimately mild uropathy. We found a partially obstructing staghorn calculus with asymmetrical cortical thinning. Left Robot-Assisted Nephro-ureterectomy plus excision of bladder cuff was planned in which 3 × 4 cm mass involving the left renal pelvis was excised. To date, there is no radiologic evidence of disease recurrence.

**Clinical discussion:**

MEST in young adults is an extremely rare tumor. They have been referred to by many alternate synonyms including ‘adult mesoblastic nephroma’ and ‘cystic nephroma’ with ‘ovarian’ or ‘cellular’ type stroma. Majority of patients with MEST present, with hematuria, abdominal pain, palpable flank mass, recurrent urinary tract infections. Similarly, our patient presented initially with nonspecific pain in the left flank region. Majority of cases in the literature presented with the tumor in benign stage, with localized spread, and without recurrence.

**Conclusion:**

Mixed epithelial and stromal tumors (MEST) of the kidney are distinct entities of benign kidney tumors. MEST in young males is a very rare entity, and a small number of cases exist. Histopathology plays a very cardinal role in diagnosis, and overall the disease has a promising outcome with conservative surgery.

## Highlights

• There are only very few cases exist in literature on MEST in young males which makes it an extremely rare entity.
• In the recent WHO classification of renal neoplasms, cystic nephroma was recognized as a separate entity, with MEST being tumors composed of a mixture of cysts, microcysts and tubules with variable stroma of cellular type.
• Histopathology plays a very cardinal role in diagnosis.
• Overall the disease has a favorable prognosis with conservative surgery.

## 1 Introduction

A Mixed epithelial and stromal tumor (MEST) refers to a complicated cystic and solid tumor, with stromal as well as epithelial components, located in the kidney. The stromal element resembles the ovarian stroma and chiefly entails the spindle cells, which contain progesterone and estrogen receptors on their surface. Epithelium-lined cysts or micro cysts mainly make up its epithelial component [1]. In the vast majority of the reported cases, MEST was found to be benign, after being resected surgically. However, a handful of cases of MEST, being associated with malignant sarcoma have also been reported [[2], [3], [4], [5], [6], [7]]. Local recurrence after surgery has also been described in a minority of reported cases [4,7]. MEST accounts for 0.2% of all renal cancers [8]. Moreover, it's predominantly found in females [9]. Its occurrence in males is quite exceptional. In this case report, we present a rare case of MEST in the kidney, presenting in a 24 years old male.

This case report has been reported in line with the SCARE Criteria [10].

## 2 Case presentation

A 24 years old, unmarried male, resident of Dera Ghazi Khan, presented at Sindh Institute of Urology and Transplantation (SIUT) with the complaint of left flank pain on and off for one month. He had a positive family history of stone disease. He had a history of stone passage and appendectomy being attempted in 2007. On examination, he was a young male of average height and built, well oriented to time, place, and person. Hematological investigation showed hemoglobin of 11.2g/dl, platelet count 280000 × 109 per liter, Total leucocyte Count (TLC) 5.2 × 109/L. Urine D/R and Urine culture were negative. Serum Creatinine was 0.88mg/dl, blood urea nitrogen was 18 and an international normalized ratio (INR) of 1.01 was found. Serologic tests for hepatitis B virus, hepatitis C, and human immunodeficiency virus (HIV) were negative. Vitals included pulse 74/min, afebrile, respiratory rate 16/min, and blood pressure was 136/78 mmHg. On General physical exam Anemia and Jaundice were negative, Lymph nodes were not palpable, and Abdomen was soft and non-tender. A Healed scar mark in the right iliac fossa was noted. No swelling was appreciated on the cough. The rest of the examination was unremarkable. Bladder histopathology report showed mild, chronic, non-specific cystitis. No evidence of dysplasia, TB, or tumor was seen. Cystoscopy and biopsy were done which showed no remarkable findings.

**Fig. 1:**
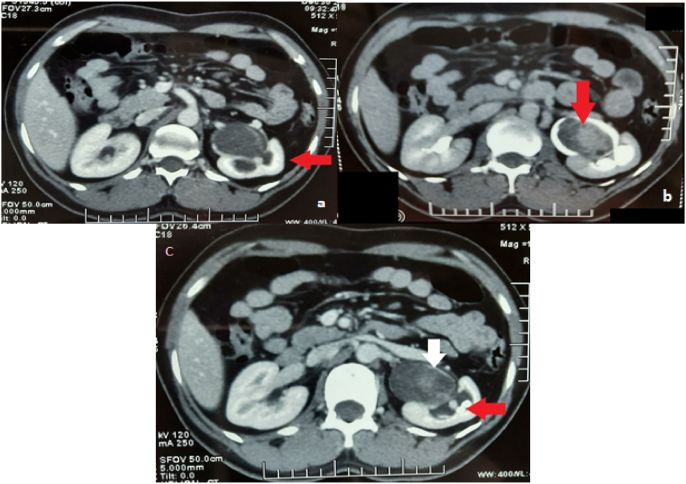
a-c Axial view of CT scan abdomen showing mild to moderate hydronephrosis (red arrow) secondary to pelvic mass (a). Axial view CT scan abdomen showing mass (red arrow) in the pelvis with peripheral enhancement (b). Axial view of CT scan abdomen showing obstruction secondary to pelvic mass (white arrow) and multiple calculi (red arrow) (c). (For interpretation of the references to colour in this figure legend, the reader is referred to the Web version of this article.)

A CT scan was done in Sindh Institute of Urology and Transplantation (SIUT) that revealed soft tissue density mass in the left renal pelvis extending down to mid-pole calyces to pleviuretric junction leading to left renal pelvis distention and obstruction. This obstruction resulted in mild to moderate uropathy on the same side (as shown in Fig. 1a). The lower pole of the kidney illustrated partially obstructing staghorn calculus with substantial asymmetrical cortical thinning. The patient was planned for a left Nephro-ureterectomy but lost to follow up.

After 2.5 years patient again presented with the same complaint. On laboratory investigation, glucose, hemoglobin, erythrocytes, creatinine, urea, liver enzymes, and cholesterol were within normal limits. CT scan Abdomen was done that showed some similar findings as previous CT scan but over time enhancing tissue density mass in left renal pelvis shifted the intensity of mild uropathy to moderate. The mass lesion increase in size since the previous scan, previously it measured 2.6 × 4.2 × 4.2cm and now it was 3.3 × 4.7 × 5.0 cm (Fig. 1b). Multiple small calculi were also noted (Fig. 1c). The larger partial staghorn calculi obstructed lower calyx.

**Fig. 2:**
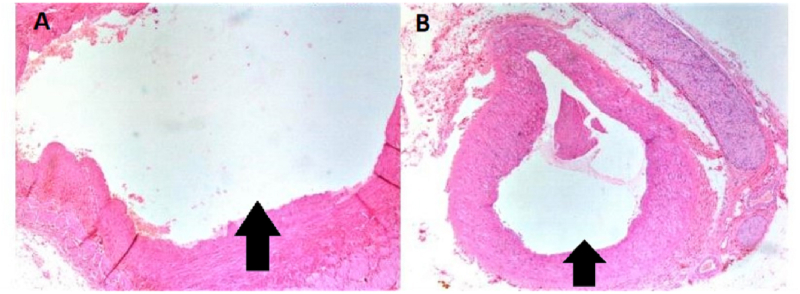
A: Hilar vessel free of tumor, B: right ureter free of tumor.

**Fig. 3:**
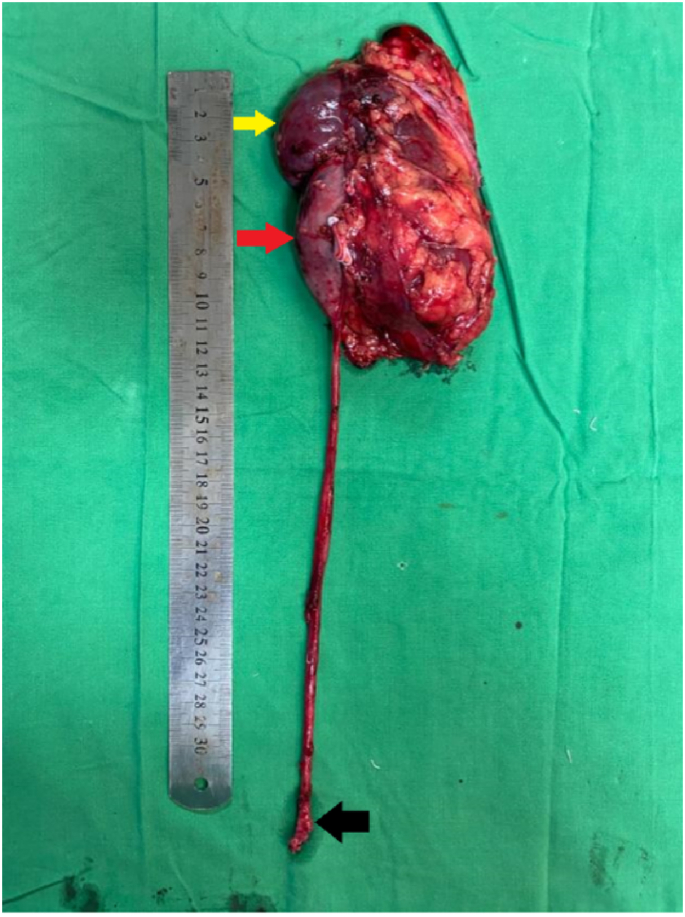
Normal renal parenchyma (yellow arrow), renal pelvis mass. (For interpretation of the references to colour in this figure legend, the reader is referred to the Web version of this article.)

**Fig. 4:**
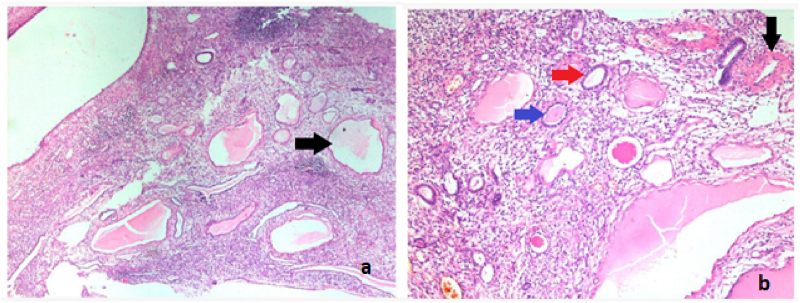
**a-b** Lower power view showing biphasic pattern i.e. stromal and epithelial components. Epithelial components show tubules and cyst (black) (a). Medium power view showing tubules (black arrow) and cysts (blue arrow) lined by a single layer of the columnar cell (red arrow) (b). (For interpretation of the references to colour in this figure legend, the reader is referred to the Web version of this article.)

**Fig. 5:**
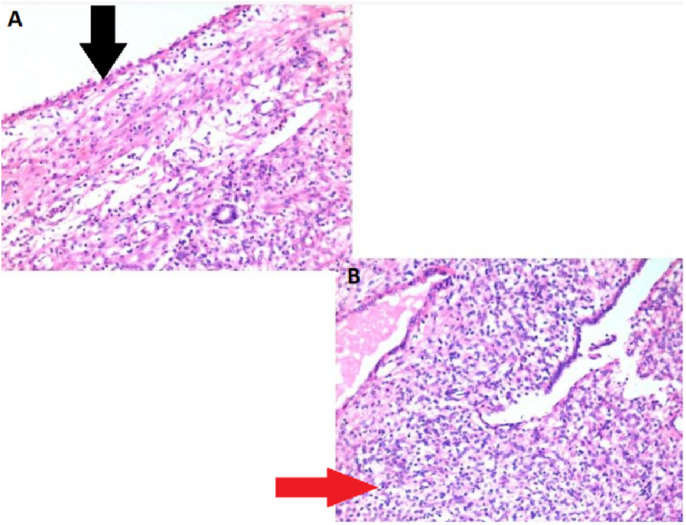
a-b High power view showing a hobnailing pattern of the epithelium (black arrow) (a). Showing stromal component with dense spindle cell (red arrow) (b). (For interpretation of the references to colour in this figure legend, the reader is referred to the Web version of this article.)

Left Robot-Assisted Nephro-ureterectomy plus excision of bladder cuff was planned in which 3 × 4 cm left renal mass involving renal pelvis was excised. Hilar vessels and the right ureter were free of the tumor as shown in Fig. 2a and b. Grossly, the mass was well-circumscribed with normal-appearing parenchyma with an obvious renal pelvis mass represented (Fig. 3). Histopathology report unveiled features of benign biphasic tumor epitomized (Fig. 4a). The epithelial component that was composed of various numbers of tubules, which ranged from very minute to larger tubules with micro and macro-cysts, lined by a single layer of columnar cells is also illustrated in Fig. 4b. It shows a hob nailing pattern of the epithelial lining (Fig. 5a). The stromal element was composed of dense spindle cells arranged in varying degrees of short bands shown in Fig. 5b. Markers (estrogen and progesterone) were positive, diffusely, and strongly present in all tumor cells. The diagnosis of mixed epithelial and stromal tumor (MEST) was then established.

Postoperatively, the patient was discharged on day 3. No other complications were observed. As it was a benign tumor no adjuvant chemotherapy was given. Now the patient is on follow-up. To date, there is no radiologic evidence of disease recurrence.

## 3 Discussion

A mixed epithelial and stromal tumor (MEST) is a rare, adult, biphasic tumor of the kidney that has an admixture of an epithelial and mesenchymal component. It was first recognized as a distinct entity by Adsay et. Al and Michal and Syrucek et. Al in 1998 [[11], [12],^12^]. They are extremely rare tumors with only a few cases reported in the literature. In past, they have been referred to by many alternate synonyms including ‘adult mesoblastic nephroma’ and ‘cystic nephroma’ with ‘ovarian’ or ‘cellular’ type stroma [12]. In the recent WHO classification of renal neoplasms, cystic nephroma was recognized as a separate entity, with MEST being tumors composed of a mixture of cysts, microcysts, and tubules with variable stroma of cellular type [13]. The tumor classically presents in perimenopausal to older women, at a mean age of 45 years as a combined solid and cystic mass. Most cases also have a history of exposure to estrogen therapy [14]. However our case was a young adult male, and so far very few cases of MEST in young males have been reported [15,16].

Majority of patients with MEST present, with urinary symptoms such as hematuria, abdominal pain, palpable flank mass, recurrent urinary tract infections, or simply as an incidental finding. A review of the literature reveals more cases presenting as incidental findings which could be explained due to advanced radiological investigations and increasing awareness amongst patients for medical checkups, both routine and non-routine [17]. Our patient also presented initially with nonspecific pain in the left flank region, as well as on a repeated visit to the hospital after failing to follow up after the first time. He came with a mass of more than 4cm, similar to previous studies that showed an average of 6cm tumor at presentation [12].

Majority of cases in the literature presented with the tumor in benign stage, with localized spread, and without recurrence [7]. Presentation of our case is also consistent with it. However malignant cases with recurrences have also been reported. Our patient, however, has been on regular follow-up for the last 6 months with no clinical or radiological evidence of recurrence.

On gross examination, the tumor in most cases reveals a variably circumscribed, encapsulated tan yellow with an admixture of solid and cystic areas, either of which may be more dominant [18]. In our case, similar macroscopic features were appreciated.

On microscopy, the tumor consists of epithelial and stromal components. The mesenchymal component presents as fascicles of spindle cells with smooth muscle, fibroblastic, and/or myofibroblastic tissue interspersed with bundles of collagen. The epithelial component is an important part of the tumor, and is not limited to the surface, and may be found within the mesenchymal tissue. In our case, the tumor showed tubules with cysts lined by columnar cells demonstrating hob nailing pattern, consistent with literature which also has evidence of simple to complex tubule-papillary pattern with or without dilatation by cysts [9]. Mitoses, pleomorphism, and necrotic changes were not demonstrated in our case, further indicating a benign disease.

Our patient also had a significant history of the passage of stones 13 years back, with a strong family history. During current management, the CT scan abdomen initially revealed two large calculi in the lower pole of a left kidney that progressed to partial staghorn calculi in the next CT scan. The calculi may be contributing to the hydronephrosis and resultant abdominal pain. Such calculi were not observed in literature so far, making this a unique finding. Jing Ye et al. proposed that MRI is a better investigation to diagnose this condition in his article, along with imaging guidelines for this particular tumor on account of its rarity [19]. However, our patient was able to be diagnosed with clinical expertise and limited investigations and managed satisfactorily.

In immunohistochemistry, the mesenchymal component shows smooth muscle differentiation as in our case, and the epithelial component-in majority of cases-shows positive estrogen and progesterone receptors, a finding strongly found in our patient after removal of tissue and study [20]. Cytogenetic studies were not warranted and hence were not performed.

Differential diagnoses of tumors that may present as MEST include cystic nephroma, as they both share multiple clinical, morphologic, histopathological, and immunohistochemical features, and Turbiner et al. proposed both of them to be unified into a single entity titled “renal epithelial and stromal tumor (REST)” [12].

## 4 Conclusion

In conclusion, mixed epithelial and stromal tumors of the kidney are a distinct entity of benign kidney tumors, which should be placed down on the differentials of a patient presenting with non-specific abdominal and/or genitourinary symptoms. MEST in young males is an extremely rare entity, and very few cases exist in literature. Histopathology plays a very cardinal role in diagnosis, and overall the disease has a favorable prognosis with conservative surgery.

## Patient perspective

The patient did not present his point of view.

## Provenance and peer review

Not commissioned, externally peer reviewed.

## Ethical approval

This is a case report that does not require a formal ethical committee approval.

## Source of funding

The authors declare that they have no funding.

## Author contribution

MTT, KSK: Study concept or design; MTT, SU, KSK, AK, IU: Data collection, data analysis or interpretation, writing the paper and IU: Critical revision of the article.

## Consent

Written informed consent was obtained from the patient for publication of this case report and any accompanying images. A copy of the written consent is available for review by the Editor-in-Chief of this journal.

## Registration of research studies

This is a case report that does not require a registration of research studies.

## Guarantor

Kiran Shafiq Khan and Irfan Ullah.

## Declaration of competing interest

The authors declare that there is no conflict of interest.
